# The Brain in Motion II Study: study protocol for a randomized controlled trial of an aerobic exercise intervention for older adults at increased risk of dementia

**DOI:** 10.1186/s13063-021-05336-z

**Published:** 2021-06-14

**Authors:** Renata L. Krüger, Cameron M. Clark, Adrienna M. Dyck, Todd J. Anderson, Fiona Clement, Patrick J. Hanly, Heather M. Hanson, Michael D. Hill, David B. Hogan, Jayna Holroyd-Leduc, R. Stewart Longman, Meghan McDonough, G. Bruce Pike, Jean M. Rawling, Tolulope Sajobi, Marc J. Poulin

**Affiliations:** 1 Department of Physiology & Pharmacology, Cumming School of Medicine, University of Calgary, Calgary, Alberta T2N 4N1 Canada; 2 Hotchkiss Brain Institute, Cumming School of Medicine, University of Calgary, Calgary, Alberta T2N 4N1 Canada; 3 Department of Physiology & Pharmacology, Cumming School of Medicine, University of Calgary, Calgary, Alberta, T2N 4N1, Canada, Calgary, Alberta Canada; 4 Department of Cardiac Sciences at the University of Calgary, Calgary, Alberta T2N 4N1 Canada; 5 Libin Cardiovascular Institute of Alberta, Cumming School of Medicine, University of Calgary, Calgary, Alberta T2N 4N1 Canada; 6 Department of Community Health Sciences at the University of Calgary, Calgary, Alberta T2N 4N1 Canada; 7 O’Brien Institute for Public Health, Cumming School of Medicine, University of Calgary, Calgary, Alberta T2N 4N1 Canada; 8 Sleep Centre, Foothills Medical Centre, University of Calgary, Calgary, Alberta T2N 4N1 Canada; 9 Seniors Health Strategic Clinical Network™, Alberta Health Services, Edmonton, Alberta Canada; 10 Department of Clinical Neurosciences at the University of Calgary, Calgary, Alberta T2N 4N1 Canada; 11 Department of Medicine at the University of Calgary, T2N 4 N1, Calgary, Alberta Canada; 12 Department of Radiology at the University of Calgary, Calgary, Alberta T2N 4N1 Canada; 13 Faculty of Kinesiology, University of Calgary, Calgary, Alberta T2N 4N1 Canada; 14 CAIP Chair in Healthy Brain Aging, Calgary, Canada; 15 Department of Family Medicine at the University of Calgary, Calgary, Alberta T2N 4N1 Canada; 16 Brenda Strafford Foundation Chair in Alzheimer Research, Calgary, Alberta Canada; 17 Heritage Medical Research Building, Room 210, 3330 Hospital Drive NW, Calgary, Alberta T2N 4N1 Canada

**Keywords:** Alzheimer’s disease, Behavioural support, Brain health, Cognitive function, Dementia, Physical activity, Sleep quality

## Abstract

**Background:**

There remains no effective intervention capable of reversing most cases of dementia. Current research is focused on prevention by addressing risk factors that are shared between cardiovascular disease and dementia (e.g., hypertension) before the cognitive, functional, and behavioural symptoms of dementia manifest. A promising preventive treatment is exercise. This study describes the methods of a randomized controlled trial (RCT) that assesses the effects of aerobic exercise and behavioural support interventions in older adults at increased risk of dementia due to genetic and/or cardiovascular risk factors. The specific aims are to determine the effect of aerobic exercise on cognitive performance, explore the biological mechanisms that influence cognitive performance after exercise training, and determine if changes in cerebrovascular physiology and function persist 1 year after a 6-month aerobic exercise intervention followed by a 1-year behavioural support programme (at 18 months).

**Methods:**

We will recruit 264 participants (aged 50–80 years) at elevated risk of dementia. Participants will be randomly allocated into one of four treatment arms: (1) aerobic exercise and health behaviour support, (2) aerobic exercise and no health behaviour support, (3) stretching-toning and health behaviour support, and (4) stretching-toning and no health behaviour support. The aerobic exercise intervention will consist of three supervised walking/jogging sessions per week for 6 months, whereas the stretching-toning control intervention will consist of three supervised stretching-toning sessions per week also for 6 months. Following the exercise interventions, participants will receive either 1 year of ongoing telephone behavioural support or no telephone support. The primary aim is to determine the independent effect of aerobic exercise on a cognitive composite score in participants allocated to this intervention compared to participants allocated to the stretching-toning group. The secondary aims are to examine the effects of aerobic exercise on a number of secondary outcomes and determine whether aerobic exercise-related changes persist after a 1-year behavioural support programme (at 18 months).

**Discussion:**

This study will address knowledge gaps regarding the underlying mechanisms of the pro-cognitive effects of exercise by examining the potential mediating factors, including cerebrovascular/physiological, neuroimaging, sleep, and genetic factors that will provide novel biologic evidence on how aerobic exercise can prevent declines in cognition with ageing.

**Trial registration:**

ClinicalTrials.gov NCT03035851. Registered on 30 January 2017

## Background

Life expectancy is projected to rise over the coming decades for most industrialized countries [1]. There are already more than 1 billion people worldwide aged 60 years or older [2]. Despite the increase in longevity, the number of years spent in good physical health (e.g,. no mobility, functional, and/or cognitive impairments) has remained unchanged over the past few years in Canada [3]. There are a growing number of older individuals with age-related normative cognitive decline as well as Alzheimer’s disease and related dementias (ADRD). Recent estimates suggest that worldwide, ~ 50 million people currently have dementia, and by 2030, this is predicted to increase to ~ 82 million [4]. In Canada, ~ 500,000 older adults are now living with dementia [5]. Almost 40% of Canadians over the age of 65 have some degree of cognitive impairment [5]. Their direct cost care was estimated to be $10.4 billion CDN dollars in 2019 and are projected to climb to $16.6 billion CDN by 2031 [5]. The personal costs of dementia and these alarming public health estimates are motivating efforts to identify lifestyle interventions that can slow the progression of ADRD. In addition to focusing on treatment, studies have been targeting the prevention of neurodegenerative diseases with the aim of mitigating, delaying, or preventing the onset of ADRD [6–10].

Exercise is a promising method to reduce the risk of dementia in both healthy older adults and those at elevated risk of ADRD due to cardiovascular risk factors [11]. The physiological benefits of exercise in older adults are clear in the research literature and include improved arterial compliance (i.e., the ability of a vessel to expand as needed), endothelial function, energy metabolism, sleep quality, and muscle mass/strength [12]. Exercise also promotes cardiovascular fitness by improving global vascular health, including increases in middle cerebral artery vasodilation responses and cerebral blood flow (CBF) [13]. These brain adaptations could play an important role in delaying the onset of ADRD as greater CBF may prevent and/or reduce the accumulation of amyloid β in the brain, which is one of the main pathological hallmarks of AD [14]. Additionally, improved cerebral haemodynamics may help prevent or slow other conditions that act as risk factors for cognitive decline and ADRD, such as cardiovascular disease (CVD) and diabetes [15]. Although steady-state CBF normally declines with post-maturation ageing, chronic diseases like CVD, hypertension, and diabetes can accelerate age-related CBF alterations and lead to disruption of neuronal homeostasis [16, 17].

Existing scientific literature suggests that high levels of physical activity can positively impact cognitive function in middle-aged (50–64 years) and older (> 65 years) individuals [18–24]. Sofi and colleagues (2011) examined 15 prospective cohort studies that collectively followed more than 30,000 healthy older adults over 1–12 years. Individuals who were more physically active before the follow-up period had a 38% reduced risk of cognitive decline compared to those with a sedentary lifestyle at baseline [22]. Cross-sectional studies have found an association between higher levels of physical activity in older adults with both better performance on specific cognitive tasks [23, 25] and reduced risk of cognitive decline [24].

Evidence from neuroimaging studies also supports the positive effects of exercise on brain health. Exercise has been shown to reduce age-related atrophy in grey and white matter [26], decrease both brain [27] and hippocampal atrophy [28], and increase white matter integrity [29, 30]. Animal models suggest changes in neurotrophic factors in response to exercise may be partially mediated by enhanced levels of brain-derived neurotrophic factor (BDNF) and insulin-like growth factor (IGF-1) in the hippocampus [31]. BDNF improves overall neural health by increasing brain vascularization, neurogenesis, and synaptic efficiency in the hippocampus [31]. As BDNF plays a role in memory formation, enhanced levels of BDNF in the brain may help prevent memory loss and cognitive decline with ageing [31].

The literature on the associations between exercise, cognition, and brain health in older individuals primarily comprised epidemiological and observational studies [22–24, 29, 30]. These study designs only allow passive observation of events and are prone to selection, information, and confounding bias compared to RCTs and cannot be used to determine causality. The few RCTs investigating the relationship between exercise, cognition, and brain health have found, however, contradictory results [12, 32–34]. Possible explanations for the conflicting literature include sub-optimal study design and methods, such as small sample sizes, short exercise interventions, and inadequate tracking of participant adherence to prescribed exercise routines [8, 34, 35]. The methodological limitations of previous studies support the need for new well-designed RCTs to investigate the association between exercise and cognitive function in older adults [21].

Most prior RCTs of the effects of exercise did not include older participants at greater risk for ADRD due to CVD and/or genetic risk factors. Both CVD and ADRD share a number of risk factors (i.e., age, obesity, physical inactivity, smoking, elevated blood pressure, and high cholesterol) [36]. Older individuals with CVD risk factors may benefit more from exercise interventions in terms of their brain health and/or cognitive performance. People who have a family history of ADRD might also gain more from exercise programmes due to their genetic susceptibility from, for example, carrying the apolipoprotein E (*APOE*) e4 allele. Individuals with at least one *APOE* e4 allele copy are at greater risk of developing AD [37].

Given the estimates of the burden of ADRD and the promising evidence of the benefits of exercise on cognitive health, we propose an RCT of aerobic exercise for individuals at increased risk of ADRD. This RCT will test the efficacy of a 6-month aerobic exercise intervention for the primary and secondary prevention of ADRD in older adults (50–80 years old). We will measure the cognitive and cerebrovascular outcomes, including vascular reactivity, vascular biomarkers, and changes in the brain structure and function using magnetic resonance imaging (MRI). A unique feature of this study is the inclusion of assessments linking vascular risk factors, neuroimaging markers, sleep, genetic risk, and cognitive health outcomes. The primary aim of this study is to determine the independent effect of aerobic exercise on a cognitive composite score, calculated as the average of ten cognitive tests (executive functioning, complex attention, processing speed, and verbal memory), between participants allocated in the aerobic vs stretching-toning groups. We hypothesize that participants randomized to the 6-month aerobic exercise intervention will score higher in the cognitive composite score following 6 months of training compared to participants allocated to a stretching-toning exercise intervention (control group). The secondary aims are to examine the comparative effects of aerobic exercise and stretching-toning on a number of secondary outcomes noted in the text and whether exercise-related changes persist 12 months after completion of the exercise intervention (i.e., at 18 months) and if a telephone-based behavioural support intervention leads to improved maintenance of the exercise-related benefits. We hypothesize that the effects of improved aerobic fitness and brain health (e.g., increase in resting CBF) seen at 6 months with aerobic exercise will be at least partially maintained at the end of the follow-up period and that the telephone-based behavioural support intervention will be more effective in leading to persistent lifestyle changes and greater retention of any benefits that arise. To test these hypotheses, the participants will be randomly allocated into one of four treatment arms: (1) aerobic exercise and health behaviour support, (2) aerobic exercise and no health behaviour support, (3) stretching-toning and health behaviour support, and (4) stretching-toning and no health behaviour support.

## Methods

The Brain in Motion II Study is an 18-month randomized controlled trial (RCT). It is open labelled with blinded evaluation superiority (*aerobic exercise superior to stretching-toning*) trial of four-armed parallel groups with a 1:1:1:1 allocation ratio. The trial utilizes a PROBE (Prospective, Randomized, Open with Blinded End-points) design [38]. The study protocol has been approved by the University of Calgary Conjoint Health Research Ethics Board (REB16-1199) and registered with ClinicalTrials.gov (NCT03035851). The RCT will be carried out according to the CONSORT and SPIRIT guidelines.

### Participants

A total of 264 male and female participants at elevated risk of ADRD between the ages of 50 and 80 will be recruited from the community in Calgary, Alberta, Canada, and surrounding areas via printed advertisements (e.g., posters and newspaper ads), media campaigns, and from physician offices through the University of Calgary Department of Family Medicine Teaching Clinics.

### Eligibility criteria

A full listing of the inclusion and exclusion criteria is presented in Table 1. This study will include inactive men and women 50–80 years of age at baseline who have subjective cognitive symptoms but no dementia and either one or more CVD risk factors for ADRD or a family history of ADRD. The study exclusion criteria are as follows: age less than 50 or greater than 80 years, current physical activity greater than 150 min per week of moderate-to-vigorous intensity physical activity, absence of CVD risk factors, presence of dementia (based on DSM-5 criteria) or severe cognitive deficits (Modified Telephone Interview for Cognitive Status (TICS-M) score < 20), presence of a developmental handicap, terminal illness (life expectancy < 1 year), non-fluency in verbal and written English, history of stroke, current participation in another clinical trial, comorbid medical or neurological illnesses (e.g., multiple sclerosis) that would confound cognitive assessments or make trial completion unlikely, and contraindication for the aerobic or stretching-toning interventions.

**Table 1:** Inclusion and exclusion criteria for the *Brain in Motion II* trial

Inclusion criteria	Exclusion criteria
• Men and women aged 50–80 years (inclusive) • Inactive (engagement < 150 min/week of moderate-to-vigorous exercise) • Subjective cognitive symptoms but no dementia^a^ • One or more CVD risk factors for ADRD or family history of ADRD^b^ • Female participants must be post-menopausal • Able to walk independently outside, as well as up and down the stairs of 20 steps	• Presence of dementia (based on the DSM-5 criteria) or severe cognitive deficits (TICS-M score < 20)^a^ • Absence of CVD risk factors or family history of ADRD^b^ • Diagnosis of severe asthma or COPD (respiratory) • Presence of a developmental handicap • Terminal illness (life expectancy < 1 year) • Not fluent in verbal and written English • History of stroke or serious cardiovascular condition • Current participating in another trial • Comorbid medical or neurological illness that would confound cognitive assessments or make trial completion and unlikely^c^ • Contraindication for exercise interventions

^a^Existing or suspected dementia will be identified by medical history, cognitive impairment on the Telephone Interview for Cognitive Status (TICS-modified; score ≤ 20), or impaired Instrumental Activities of Daily Living (IADL)—a response of needs assistance or dependent due to cognitive impairments on any item on the Lawton scale

^b^Cardiovascular disease (CVD) risk factors for Alzheimer’s disease and related dementias (ADRD) include the history of hypertension, diabetes mellitus, obesity (body mass index (BMI) BMI ≥ 30 but < 40 kg/m^2^), elevated cholesterol, current smoking, and history of coronary artery disease without recent (< 5 years) symptoms. Family history of ADRD is defined as having a first-degree relative (parent, sibling, or child) who has been diagnosed with ADRD

^c^E.g., persistent post-concussive symptoms or medication for psychological condition that would impact cognitive performance

Inactivity will be assessed with a physical activity questionnaire [39] and defined as an engagement in < 150 min/week of moderate-to-vigorous exercise [40]. The subjective cognitive symptoms, family history of dementia, CVD risk factors for ADRD, family history of ADRD, and current physical activity level will be assessed during a telephone interview. The aim is to recruit participants with either subjective cognitive decline or mild cognitive impairment who are at risk for progressive cognitive decline and dementia. This recruitment strategy is similar to the one used in a previous RCT that showed that exercise reduced the likelihood of cognitive decline [41], as measured by the Clinical Dementia Rating Scale [42]. That scale has been validated [43] and allows, with reasonable accuracy, the identification of individuals with mild cognitive impairment and those with very early AD. The presence of suspected dementia will be identified by medical history and cognitive impairment on the Telephone Interview for Cognitive Status (TICS-modified; score ≤ 20 [44, 45];). The CVD risk factors for ADRD include a history of hypertension, diabetes mellitus, obesity (body mass index (BMI) < 40 kg/m^2^), elevated cholesterol, current smoking, and coronary artery disease without recent (< 5 years) symptoms. A family history of ADRD is defined as having a first-degree relative (parent, sibling, or child) who has been diagnosed with ADRD.

Following the telephone evaluation, the participants will be provided with a copy of the informed consent form to review—prior to the first one-site visit—as part of a “welcome package” and will also be asked to provide written permission to contact their family physician for medical clearance to participate in the study. The study coordinator will be responsible for obtaining the informed consent. Participants will then attend a 60-min on-site clinical laboratory eligibility screening session in which a written informed consent will be obtained by the study coordinator. During this visit, the participants will also complete the following assessments: the Brain Injury Screening Questionnaire (BISQ [46];), the Montreal Cognitive Assessment (MoCA [47];, the Memory Assessment Clinic questionnaire (MAC-Q [48];), and the Physical Activity Readiness Questionnaire (PAR-Q+ [49, 50];). The BISQ is a screening tool used to identify a lifetime history of traumatic brain injury [46]. The MoCA score will be used to stratify the participants at baseline prior to their allocation into different experimental groups (see below). The MAC-Q is a validated measure of subjective memory complaints that has been previously used in healthy older and clinical/research populations [51]. Finally, the PAR-Q+ form will be used to determine the safety of exercise programme participation. This form will be completed by the participants and included in a fax sent to the participant’s family physician requesting medical clearance to participate in the study. A certified exercise physiologist will review this questionnaire with participants prior to completing their exercise testing. Additionally, participants will be requested to complete a sociodemographic questionnaire and report on their use of medications and other therapies.

### Risks

Risks of exercise include falls, musculoskeletal injuries, coronary heart disease events (myocardial infarct, acute coronary syndrome), and other side effects that are generally minor. Our previous experience with similar exercise interventions gives our team considerable expertise on the means to mitigate these types of health risks. Maximal cardiopulmonary testing will be conducted by certified exercise physiologists with a physician on-call and present for high-risk participants and appropriate emergency equipment available. A participant may be withdrawn from the study if a research procedure is judged to be potentially harmful or presents an unacceptable risk of injury to the participant. For example, participants could be withdrawn if they develop an abnormality (e.g., abnormal ECG suggesting an unstable cardiac issue, uncontrolled hypertension) that is judged by a physician associated with the study or the participant’s attending physician to pose a risk to the participant if they continue in the study.

### Sample size

Preliminary estimates of the effect sizes for improvement in overall cognition (cognitive composite score) with aerobic exercise training are based on the results of a previous study from our group, the Brain in Motion Study [52], that used similar active intervention and outcome. This study will recruit a total of 264 participants. This power calculation, which is based on a sample size calculation formula for a mixed model for repeated measures data with attrition [53], provides 80% power to detect 0.4 standard deviation between-group difference for an average repeated measurement correlation of 0.6 and three repeated measurements, taking 20% attrition into consideration (based on the *Brain in Motion* Study [52];).

### Randomization

This study will be a prospective open label with blinded evaluation (PROBE) trial and randomize the participants into one of the following four treatment arms:

1. Aerobic exercise and health behaviour support: Participants will undergo aerobic exercise training for 6 months and will receive 1-year of individually tailored telephone support after the intervention.
2. Aerobic exercise and no health behaviour support: Participants will undergo aerobic exercise training for 6 months and will not receive health behaviour support during the follow-up 1-year follow-up period (at 18 months).
3. Stretching-toning and health behaviour support: Participants will undergo stretching-toning exercise training and will receive 1-year of individually tailored telephone support after the intervention.
4. Stretching-toning and no health behaviour support: Participants will undergo stretching-toning exercise training and will not receive health behaviour support during the follow-up 1-year follow-up period (at 18 months).

Randomization sequence within blocks will be generated by computer using a random number generator. Randomization will be stratified by age (age > 65 vs ≤ 65 years) and sex with blocked, simple randomization into each of 4 strata for each group of participants as they enter the study. Because enrolment occurs in complete groups of participants (rather than sequentially), randomization will be done in complete blocks such that randomness of allocation is completely preserved. Specifically, recruitment for each study site (e.g., University of Calgary, YMCA Calgary) will be done independently. Once the desired number of participants (8–15 participants) are recruited, baseline testing will be conducted. Participants will be then randomized (the random sequence will be generated by computer: REDCap randomization tool) and immediately start the exercise intervention. Randomized allocation will be completely masked to all study personnel, except the database programmer (study coordinator) who will not participate in any participant or protocol-related activities. Participants will receive a “welcome package” containing their allocation group during the baseline testing visits, in advance of the exercise programme starting date.

### Blinding

The nature of both the aerobic exercise intervention and health behaviour intervention precludes participants from being blinded to the group status; however, the researchers assessing any of the outcome variables will be blinded to participant group assignment to avoid bias in result interpretation. Since patients are not blinded, safety can be assessed in the context of the actual intervention, and, therefore, the research team will remain blinded to the group allocation.

### Study design

**Fig. 1:**
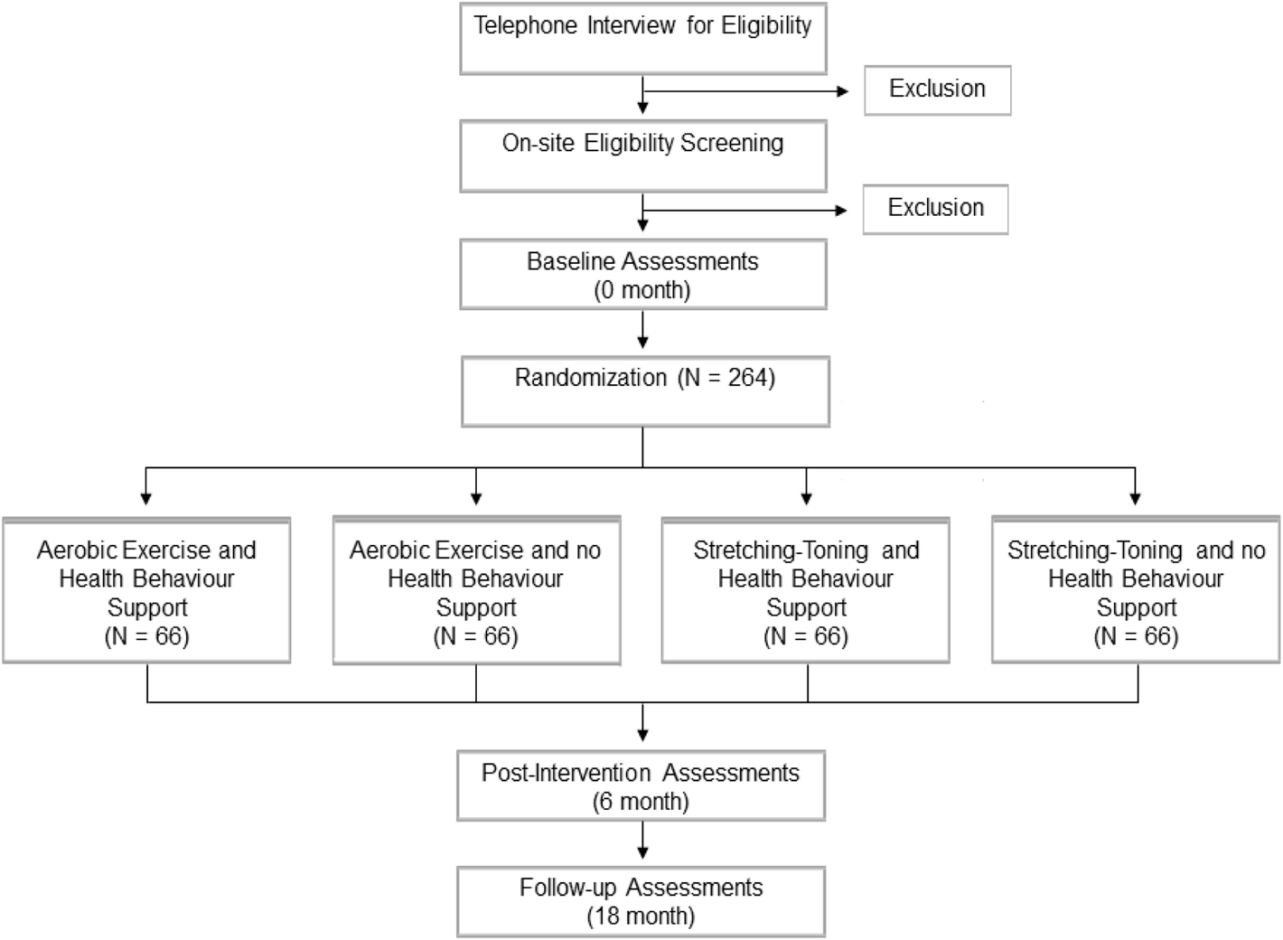
Overview of study design and participant flow through the trial

Figure 1 displays an overview of the study design and participant flow through the trial. Once a participant is eligible to join the study, he or she will be asked to provide additional information during the 60-min on-site eligibility screening session, including basic socio-demographic information (e.g., sex and gender), medical history (cardiovascular, respiratory, neurological), lifestyle habits (e.g., smoking history, physical activity levels), descriptive physical data (e.g., height, weight), level of education, current and past income and occupation, and current medications (prescribed, over-the-counter, and vitamins or supplements).

After the completion of the screening procedures, the participants will be randomly assigned to one of the four experimental groups. Following randomization, participants will undergo their first set of cerebrovascular/physiological, neuroimaging, sleep, and cognitive factors and will complete several lifestyle and psychological questionnaires (see Table 2). This battery of assessments will be completed again following the 6-month intervention period and after a 1-year follow-up period.

**Table 2:** Schedule of physiological, cognitive, psychological, and neuroimaging assessments for the *Brain in Motion II* trial

Assessment measure	Screening assessments	Visit (months)			
		0	3	6	18
**Screening**					
Telephone Interview for Cognitive Status – Modified (TICS-m)	✓				
Montreal Cognitive Assessment (MoCA)	✓				
Medical history	✓				
Demographics and health history	✓				
Eligibility screening questionnaire	✓				
Physical Activity Readiness Questionnaire (PAR-Q+)	✓				
Brain Injury Screening Questionnaire (BISQ)	✓				
Sociodemographic questionnaire	✓				
Vital signs		✓	✓	✓	✓
**Cognition**					
Neuropsychological test battery		✓		✓	✓
**Cerebral blood flow**					
Transcranial Doppler ultrasound		✓		✓	✓
**Maximal aerobic oxygen uptake**					
Maximal aerobic capacity test		✓		✓	✓
**Blood biomarkers**					
Blood work		✓		✓	✓
**Genetic risk factors**		✓			
**Risk/protective factors**					
Global Physical Activity Questionnaire (GPAQ)		✓		✓	✓
Diet History Questionnaire		✓		✓	✓
Memory Assessment Clinic Questionnaire (MAC-Q)	✓	✓		✓	✓
Multiple Ability Self-Report Questionnaire (MASQ)		✓		✓	✓
Lifetime Cognitive Activities Questionnaire		✓			
Current Cognitive Activities Questionnaire		✓		✓	✓
Clinical Dementia Rating Scale (CDRS)		✓			✓
Lubben Social Network Scale		✓		✓	✓
Family and Friend Support for Exercise		✓		✓	✓
Physical activity-related autonomy support, persuasion, and pressure		✓		✓	✓
Motivation for leisure time physical activity (BREQ-3)		✓		✓	✓
Adult ADHD Self-Report Questionnaire (ASRS)		✓		✓	✓
Hospital Anxiety and Depression Scale (HADS)		✓		✓	✓
Geriatric Depression Scale (GDS)		✓		✓	✓
Centres for Epidemiological Studies Depression Inventory		✓		✓	✓
Beck Anxiety Inventory (BAI)		✓		✓	✓
Perceived Stress Scale Questionnaire (PSS)		✓		✓	✓
Anxiety Sensitivity Index (ASI)		✓		✓	✓
**Sleep quality**					
Polysomnography and actigraphy		✓		✓	✓
Sleep quality/daytime sleepiness/sleep disorders		✓		✓	✓
Pittsburgh Sleep Quality Index (PSQI)		✓		✓	✓
Epworth Sleepiness Scale (ESS)		✓		✓	✓
STOP-Bang		✓		✓	✓
Insomnia Severity Index (ISI)		✓		✓	✓
Restless Legs Questionnaire		✓		✓	✓
**Health behaviour**					
Motivational Readiness Questionnaire		✓		✓	✓
Exercise Benefits/Barriers Scale		✓		✓	✓
EuroQoL Five-Dimension Quality of Life Scale (EQ-5D-5L)		✓		✓	✓
Exercise Self-Efficacy Scale (EXSE)		✓		✓	✓
Adherence Intent and Self-Regulatory Processes Scale		✓		✓	✓

### Exercise interventions

Exercise sessions will be conducted in a small group (8–15 participants) by supervised by qualified trainers in community gyms and recreation centres (e.g., University of Calgary, YMCA Calgary). Costs associated with travelling will not be covered, but parking will be paid for all the exercise sessions. Trainers will have a background in kinesiology and/or certification as a personal trainer and will be trained with suitable emergency equipment and procedures in place. In the event that any of the assessments identify any concerns, the participant’s family physician will be contacted to help in any further follow-up or the appropriate referral. There will be no compensation to those who suffer harm from trial participation. Participation is voluntary, and participants may withdraw at any time and not suffer any disadvantage or reprisal for withdrawing. The researcher may request a participant to withdraw from the study if a research procedure is judged to be potentially harmful to the participant.

Participants will be asked to maintain a minimum of 80% attendance rate at the exercise sessions. Participants who miss a supervised exercise session will be encouraged to complete the session independently and record the unsupervised session in a personal workout logbook (reviewed regularly by trainers). Participants are asked to complete a fourth session each week on their own in addition to the three supervised sessions. Participants can continue with their normal daily activities, such as taking prescribed medications, and activity pattern. This information will be captured in questionnaires (administered at the beginning, during, and completion of the study), logbooks (to record any additional independent unsupervised exercise), and actigraphy (which is used to track physical activity and sleep). At the end of the exercise interventions, no instructions or stipulations will be provided for continuing to exercise, but those in the health behaviour group are provided with telephone support to help them achieve their individual physical activity and exercise goals.

#### Aerobic exercise intervention

Participants randomized to this condition will take part in a 6-month aerobic training programme held 3 days per week. Each exercise session will comprised a 5-min warm-up, 20–40 min of aerobic exercise (walking or jogging), and 5-min cool-down and stretching. Individualized exercise prescriptions will follow the current principles and guidelines established by the American College of Sports Medicine (ACSM) and the American Heart Association (AHA [40]). As participants progress through the 6-month intervention, the duration of exercise will increase from 20 min (month 1) to 30 min (months 2 and 3) and to 40 min (months 4 to 6), with proportional increases to the corresponding warm-up and cool-down periods. Exercise intensity will be based on an individual’s heart rate reserve (HRR) calculated by subtracting the resting heart rate from the maximal heart rate achieved during the incremental treadmill test conducted at baseline. Prescribed intensity will build from 30 to 45% of HRR (months 1 to 3) to mitigate the risk of injury and will progress to 60–70% HRR (months 4 to 6). Polar® heart rate monitors will be used to check compliance with target heart rate zones. Heart rate data will be exported for analysis after each exercise session.

#### Stretching-toning intervention

Participants randomized to this condition will meet on a similar schedule to that of the aerobic exercise intervention group, but the training sessions will focus on stretching and toning exercises. This programme consists of balance work, stretching, and core activation exercises and other basic movement patterns (e.g., static lunges, walking dips, and squats). Based on prior RCTs of similar interventions, we expect this control to be ineffective or minimally effective for some measures of executive function and memory compared to aerobic exercise [32]. We anticipate that stretching and toning exercises will provide a good comparison condition to aerobic exercise by maintaining participant’s enthusiasm and motivation.

### Health behaviour support follow-up

Building on well-supported theoretical foundations [54], participants randomized to these groups will undergo a 1-year of individually tailored telephone support to facilitate and maintain behaviour change [55]. Given that no “best” strategy exists for increasing physical activity levels [56], this behavioural support intervention will use an integrated model of modifiable determinants of physical activity behaviour to address intra- and inter-personal determinants [54, 57]. Support provided will address one or more constructs within the domains of cognitive, behavioural, and social support, with the specific behaviour change strategy determined by discussion with participants. Cognitive strategies target peoples’ thoughts and perspectives surrounding exercise behaviour and include techniques such as inquiring about individuals’ perceptions of the reasons underlying their motivation, intentions, and attitudes/beliefs. The behavioural domain will target strategies to alter exercise behaviour, focusing on developing skills to initiate or cue exercise behaviour and/or reduce counterproductive behaviours, including strategies such as goal setting, stimulus control, action planning, and coping planning. Finally, social strategies emphasize interpersonal resources and barriers and focus on seeking and leveraging interpersonal resources and social environments to encourage exercise, such as seeking group and/or partner exercise settings, emotional and/or informational social support, and modelling.

Participants allocated to receive support will be contacted by telephone to take part in a 15–30-min support discussion with a trained study staff member. Because early repetition of healthy behaviours is thought to precipitate larger increases in automaticity and habit formation [58], participants will receive four support phone calls within the first half of the behaviour support intervention and two in the second half.

### Primary outcome

The primary outcome will be assessed at baseline and 6 and 18 months in the research lab by personnel blinded to which group the participant was randomized. Due to the length of the study, the outcomes measured at 6 and 18 months will likely be conducted by a different person who will be trained and validated on all study procedures they administer.

#### Cognitive composite score

The primary outcome of interest is an overall cognitive composite score measured after the completion of the exercise intervention (i.e., at 6 months). Participants allocated to the aerobic exercise intervention will be compared to those allocated to the stretching-toning exercise one. The cognitive composite score incorporates five cognitive domains and their respective tests listed as follows:

1. Executive function: verbal fluency, Trail Making Test – part B (TMT-B)
2. Visual perceptual skills: Line Orientation, and Card Rotations Test
3. Complex attention: Digit and Sustained Attention to Responding Task (SART)
4. Processing speed: Symbol-Digit Modalities Test (oral and written; SDMT-O/-W) and the Trail Making Test – part A (TMT-A)
5. Memory: Hopkins Verbal Learning Test (HVLT) and Brief Visuospatial Memory Test – Revised (BVMT-R)

Raw scores of each cognitive test will be transformed to a common metric based on standard scores (Z scores). Z scores will be calculated by subtracting the normative mean (retrieved from prior studies or observations) from each participant’s component test score [59]. This difference will then be divided by the standard deviation of the normative sample [59]. Z scores from each cognitive test will be summed (i.e., each domain will be equally weighted), averaged within each of the five domains to obtain a domain score, and then compared statistically across the two interventions (aerobic vs stretching-toning) [60]. Apart from considering the overall cognitive composite score, specific domains hypothesized to be most sensitive to the aerobic exercise intervention (e.g., executive functioning and processing speed) will be analysed as secondary outcomes.

### Secondary outcomes

The secondary outcome will be assessed at baseline and 6 and 18 months in the research lab (except for sleep studies, which will be conducted at the participant’s home) by personnel blinded to which group the participant was randomized. Due to the length of the study, outcomes measured at 6 and 18 months will likely be conducted by a different person who will be trained and validated on all study procedures they administer.

#### Cognition

Apart from considering the overall cognitive composite score, specific domains hypothesized to be most sensitive to the aerobic exercise intervention (e.g., executive functioning and processing speed) will be analysed as secondary outcomes. The specific neuropsychological tests were chosen based on their relevance to ADRD and the component domains known to be affected by aerobic fitness training (executive functioning, complex attention, processing speed, and verbal memory [34, 61–63]) (see Table 3 for a complete listing of the individual neuropsychological tests that will be administered).

**Table 3:** Neuropsychological tests administered for the *Brain in Motion II* trial

Neuropsychological test by domain	Description (approximate time in min)
**Premorbid intellectual ability**	
The Spot-the-Word Test	Silent lexical decision task; pairs of items comprising one word and one non-word are presented (5 min)
**Cognitive screening**	
Montreal Cognitive Assessment (MoCA)	Brief cognitive screening tool, used to stratify and characterize the participants at baseline (10 min)
**Complex attention**	
Digit	Sequence of number strings (2–8 digits in length) presented for recall in the same order or backwards (8 min)
Sustained Attention to Responding Task (SART)	Computer-based task, withholding key presses to infrequent and unpredictable stimuli amid 225 target stimuli (8 min)
**Processing speed**	
Symbol-Digit Modalities Test (oral and written; SDMT-O/-W)	Written and oral speeded task matching digits with geometric symbols according to a legend (5 min)
Trail Making Test – part A (TMT-A)	Timed test of visuo-motor sequencing and scanning; speed of performance linked to functional outcomes (e.g., driving ability; 3 min)
**Language**	
Boston Naming Test – short version (BNT)	Naming vocabulary test; 15 pictures presented sequentially (5 min)
Token Test for Receptive Language	Identify and manipulate 20 tokens of varying size, shape, and colour in response to given directions (7 min)
**Verbal memory**	
Hopkins Verbal Learning Test (HVLT)	Twelve-word list presented for 5 trials with immediate recall, cured recall, multiple choice recognition, and delayed recall measured (14 min)
Brief Visuospatial Memory Test – Revised (BVMT-R)	Draw and recognize geometric figures that were presented 25 min earlier (10 min)
**Executive function**	
Verbal fluency	Generation of unique words beginning with designated letters (7 min)
Trail Making Test – part B (TMT-B)	Timed test of visuo-motor sequencing and scanning; performance speed linked to functional outcomes (3 min)
Judgement of Line Orientation	Matching the angle and orientation of 2 angled lines to a set of 11 lines arranged in a semi-circle (10 min)
Card Rotations Test	Identify which of six irregularly shaped and rotated cards are the same as a target card (4 min)

#### Cerebral blood flow

A CBF test will be conducted to investigate whether aerobic exercise enhances brain health (e.g. increase resting CBF) and, if yes, whether increased resting CBF due to training will be maintained over 1 year after the exercise intervention completion. Cerebrovascular responses to increases in arterial partial pressure of carbon dioxide (CO_2_) and to submaximal exercise (measures of cerebrovascular reserve) will be assessed using transcranial Doppler ultrasound. The experimental set-up and protocol used are described in detail in a previous publication from our laboratory [52]. Outcome measures for these tests include baseline CBF and cerebrovascular reserve (i.e., brain responses to CO_2_ and exercise), heart rate, blood pressure, and blood rheology (i.e., haematocrit, viscosity, and aggregation).

#### Maximal aerobic oxygen uptake

A protocol to assess maximal oxygen uptake (V̇O_2_max) will be performed to test the hypothesis that aerobic exercise will enhance aerobic fitness as measured by V̇O_2_max and that the V̇O_2_max improvement due to training will be maintained over 1 year after completion of the exercise intervention. The V̇O_2_max test will be conducted on a treadmill and will involve a ramp increase in workload aimed at reaching the subject’s V̇O_2_max within 8–12 min, according to ACSM recommendations [64]. Exercise testing will be completed in the Clinical and Translational Exercise Physiology Laboratory, Cumming School of Medicine, University of Calgary by certified exercise physiologists (Canadian Society of Exercise Physiology). The experimental set-up and the protocol used are similar to those described in previous studies from our lab (see [65]). Outcome measures for this test include V̇O_2_max, ventilatory thresholds, heart rate, and blood pressure.

#### Blood biomarkers

Blood markers will be analysed to examine if they moderate the observed effects of aerobic exercise on cognitive outcomes. We hypothesize aerobic exercise will promote more favourable changes in the blood biomarkers than stretching-toning exercise, and changes in blood biomarkers in the aerobic exercise group will be associated with cognition enhancement. Sex steroid hormone status (estradiol, progesterone, testosterone, and sex hormone-binding globulin), lipids (cholesterol, high- and low-density lipoprotein, and triglycerides), thyroid (thyroid-stimulating hormone), renal (creatinine), hepatic (alanine aminotransferase and bilirubin), and cardiovascular disease markers (hsCRP) will be measured. Complete blood count will be quantified immediately after blood collection, while other markers will be assessed in batches after blood samples are centrifuged and frozen at −80 °C. Details of the blood volumes required and assays to be used (including reliability, validity, and coefficients of variation) are included in Table 4.

**Table 4:** Details of the blood volume required, assays to be used, intra-assay variability, and measuring range

Markers	Collection tubes	Assay	Sample volume	CV% intra-assay	Measuring range
**Hormones**					
Estradiol	PST/SST	Electrochemiluminescent immunoassay	500 μL plasma/serum	6	8.4–15,781 pmol/L
Progesterone	PST/SST	Chemiluminescent immunoassay	200 μL plasma	6	0.48–190.8 nmol/L
Testosterone	PST/SST	Chemiluminescent immunoassay	0.5 mL plasma/serum	9	0.35–52.1 nmol/L
Free Testosterone	Calculated				
SHBG	SST	Chemiluminescent immunoassay	0.5 mL serum	7	0.02–180 nmol/L
Albumin	PST/SST	Bromcresol purple binding/colorimetric	0.2 mL plasma/serum	2.5	1–100 g/L
**Lipids**					
Cholesterol (total)	PST/SST	Enzymatic colorimetric—cholesterol esterase and cholesterol oxidase	0.2 mL plasma/serum	1.7	0.08–20.8 mmol/L
LDL	Calculated				
HDL	PST/SST	Enzymatic colorimetric—PEG-modified cholesterol esterase and cholesterol oxidase	0.2 mL plasma/serum	2.5	0.10–3.12 mmol/L
TG	PST/SST	Hydrolysis of TG by a lipoprotein lipase to glycerol, followed by oxidation to form hydrogen peroxide	0.2 mL plasma/serum	2.5	0.10–11.40 mmol/L
**Thyroid**					
TSH	PST/SST	Two-site chemiluminescent immunoassay (Siemens Centaur reagent)	1.0 mL plasma/serum	5.0	0.01–150 mU/L
**Kidney**					
CBC	EDTA	Beckman-Coulter GEN-S (Calibrated with SCAL kit)	4 mL whole blood		
Creatinine	PST/SST	Enzymatic colorimetric—creatininase	0.2 mL plasma/serum	2.0	5–3000 μmol/L
**Hepatic**					
ALT	PST/SST	Rate UV without pyridoxal phosphate activation	0.2 mL plasma/serum	3.0	4–600 U/L
Bilirubin (total)	PST/SST	Colorimetric (diazonium ion, with blank)	0.2 mL plasma/serum	3.0	2–600 μmol/L
**CVD marker**					
hsCRP	PST/SST	Particle enhanced turbidimetric assay	0.2 mL plasma/serum	2.0	0.1–20 mg/L

*Notes*: All analyses will be carried out at Calgary Laboratory Services. At baseline (0 month), 27–30 mL of blood will be required for measurements of hormones, lipids, and screening markers of thyroid, kidney, hepatic function, and hsCRP (3 × 5 mL gold (SST tubes) + 3 × 4 mL lavender (EDTA tubes)). At the other time points (6 and 18 months), only 18–20 mL of blood will be required for measurements of hormones, lipid profiles, and hsCRP (2 × 5 mL gold (SST tubes) + 2 × 4 mL lavender (EDTA tubes))

*Abbreviations*: *PST*, plasma separator tube; *SST*, serum separator tube; *EDTA*, ethylenediaminetetra acetic acid in a tripotassium or disodium base; *CV%*, coefficient of variation; *SHBG*, sex hormone binding globulin; *LDL*, low-density lipoprotein; *HDL*, high-density lipoprotein; *TG*, triglycerides; *TSH*, thyroid-stimulating hormone; *CBC*, complete blood count; *ALT*, alanine aminotransferase; *CVD*, cardiovascular disease; *hsCRP*, high-sensitivity C-reactive protein

#### Genetic risk factors

DNA samples will be collected at baseline to test the hypothesis that genetic risk factors moderate exercise-related cognitive outcomes. We hypothesize aerobic exercise will promote greater cognitive enhancement in those with greater changes in neurotrophic factors (e.g., BDNF and IGF-1) compared to stretching-toning exercise. Genomic DNA will be obtained from buffy coat blood samples (Gentra Puregene Blood Kit; Qiagen, Venlo, Netherlands). DNA samples will be sent for polymerase chain reaction amplification and Sanger sequencing (BigDye v1.1 Cycle Sequencing Kit; Applied Biosystems, Foster City, CA) on ABI 3130XL Genetic Analyzer (Applied Biosystems). These techniques will be used to genotype selected genes that have shown to influence cognitive performance and that are associated with neuronal integrity. These genes include BDNF, APOE, IGF-1, catechol-*O*-methyl-transferase (COMT), angiotensin-converting enzyme (ACE), insulin-degrading enzyme (IDE), methylenetetrahydrofolate reductase (MTHFR), clusterin (CLU), complement component (3b/4b) receptor 1 (CR1), bridging integrator 1 (BIN1), phosphatidylinositol-binding clatherin assembly protein (PICALM), 3-hydroxy-3-methylglutaryl-CoA reductase (HMGCR), and translocase of outer mitochondrial membrane (TOMM40).

#### Risk/protective factors

Self-administered validated questionnaires will be used to quantify the role of additional lifestyle factors on cognitive functioning at baseline, and changes over the intervention and follow-up periods. Measures include changes in dietary intake, food frequency, supplement intake [66], physical activity [67, 68], motivation for physical activity [69], cognitive activities [62], mood changes [70, 71], social support [72–74], social engagement [72, 75], and modifiable ADRD risk/protective factors [76], including attention deficit hyperactivity disorder (ADHD) [77]. These data will provide collective insights on the possible mechanisms by which our interventions improve cognitive functioning and help to prevent ADRD [76].

#### Brain structure and function

Brain MRI will be used to test the hypotheses that 6 months of aerobic training, but not stretching-toning training, is associated with the following: (1) increases in brain volume, specifically cortical grey and white matter volume, including the frontal lobes and cortical areas implicated in attention control and memory processes [27] and hippocampal volume [78]; (2) increases in MRI measured resting CBF [79]; (3) reduced progression of white matter hyperintensities of presumed vascular origin; and (4) increases in functional connectivity of the default mode network [80].

Neuroimaging data will be collected on the 3-T scanner (General Electric Discovery 750, GE Healthcare, USA). Our 37-min MRI protocol is built on the multi-site Alzheimer Disease Neuroimaging Initiative protocol [81]. Our protocol (see Table 5) includes a high-resolution whole-brain 3D T1-weighted structural image, a T2-weighted fluid-attenuated inversion recovery (FLAIR) image to evaluate white matter hyperintensities, resting perfusion measured with arterial spin labelling (ASL), high angular resolution diffusion imaging to calculate mean diffusivity and fractional anisotropy and for tractography analysis, and resting-state blood oxygen level-dependent (BOLD) functional MRI for functional connectivity analyses. Further, we will generate cerebrovascular reactivity maps from BOLD and ASL time series acquired during hypercapnia [82–87]. It is expected that 60–70% of participants will consent to the MRI component of the study; however, participants with contraindication for an MRI exam will not be included in this part of the study. Therefore, an additional 10–20% non-completion rate is expected for participants who do consent to an MRI.

**Table 5:** MRI acquisition parameters

Sequence	Repetition time (ms)	Echo time (ms)	Voxel size (mm3)	Band-width (kHz)	Others	Scan duration (min:s)
T1w (inversion-recovery prepared fast spoiled gradient echo)	6.7	2.9	1.0 × 1.0 × 1.0	31.25	Inversion time = 650 ms, 2× acceleration	5:31
T2-FLAIR	10,000	140	0.9 × 0.9 × 3.0	31.25	Inversion time = 2250 ms, 3 segments	5:01
Diffusion (spin-echo echo planar imaging)	8000	65	2.2 × 2.2 × 2.2	250.00	2× acceleration, 30 directions b-value = 1000, 3 b-value = 0 images	4:32
BOLD (echo planar imaging): resting state and CVR	2286	30	3.5 × 3.5 × 3.5	83.33	2x acceleration, 5:00 rest, 2:00 hypercapnia, 1:11 rest	8:11
pCASL (fast spin echo stack-of-spirals): resting perfusion	4786	10.2	3.6 × 3.6 × 5.0	62.50	Label duration = 1500 ms, post-label delay = 2025 ms, 2 averages	3:21
Dual echo pCASL: simultaneous BOLD and perfusion CVR	4000	12.7/28	3.75 × 3.75 × 3.8	250.00	Label duration = 1600 ms, post-label delay = 1000 ms, 2:16 rest, 2:00 hypercapnia, 2:00 rest	6:16

*Abbreviations*: *FLAIR*, weighted fluid attenuated inversion recovery; *BOLD*, resting-state blood oxygen level dependent; *CVR*, cerebrovascular reactivity; *pCASL*, pseudo-continuous arterial spin labelling

#### Sleep quality

We will test whether the beneficial effect of aerobic exercise on cognitive functioning is modulated by an improvement in sleep quality. We will use three complementary modalities to monitor sleep quality. First, we will apply the Pittsburgh Sleep Quality Index (PSQI) Questionnaire [88], which assesses the participants’ quality of sleep during the previous month. Second, we will measure inactivity as a proxy for sleep using actigraphy [89]. Finally, we will analyse sleep quality through an in-home-based overnight level two polysomnography (Embletta MPR PG, Natus Medical Inc., Pleasanton, CA) and an ST1 proxy unit.

#### Cost-utility analysis

To estimate the cost-utility of our aerobic exercise intervention, we will assess costs associated with the intervention itself (e.g., recreational facility membership, personal trainer time, equipment), physical activity engaged in during the maintenance phase, and healthcare resource utilization. Using provincial administrative databases, costs of pharmaceuticals, primary care physician visits, emergency room visits, and hospitalizations will be calculated. We will use the EuroQoL Five-Dimension Five-Level Quality of Life Scale (EQ-5D-5L [90–92];) to measure the quality of life. The scores will be translated into utilities using the Canadian social value set [93].

### Data management and monitoring

This study will be conducted in a manner consistent with good clinical practice. Drs. MJP, MDH, and DBH will oversee all research activities. The study coordinator will be responsible, in part, for the communication with all investigators. The team leader, project manager, and site coordinator will hold weekly meetings; full team meetings will be held monthly.

The data will be collected in paper forms, recorded in secured and encrypted electronic report forms (REDCap) by trained personnel, and verified by an independent trained staff member. Once the data is verified, the documents will be scanned and uploaded to REDCap. The data storage will be in secure servers requiring two-factor authentications. All the paper forms (screening and demographic data and outcome measures) will be recorded by participant number and stored in locked filing cabinets, to which only the study coordinator and primary investigator possess a key, after verifications.

Investigators will maintain data and records on file for a minimum period of 25 years. These will include the enrolment records, receipts, undertakings signed by the qualified investigator in this study, copies of the protocol, and attestation by the Research Ethics Board that our study carries out its functions in a manner consistent with good clinical practice.

Amendments in the research protocol, including consent form changes, will be communicated to investigators and local REB. Individuals already enrolled in the study will be asked to sign an updated version of the consent form if applicable.

This RCT does not require regulation by Health Canada (no Clinical Trial Application is sought). Serious adverse event (SAE) information will be collected for the duration of the participant’s involvement in the RCT. SAEs will be managed according to the best current standard of care and reported to local REB according to good clinical practices. All SAEs will be reported within one business day, in a structured narrative explaining the events that occurred. An internal safety monitor will adjudicate all SAEs for report completeness, seriousness of event, and relationship to study interventions.

#### Data withdrawal

Participants may request the removal and permanent deletion of data and/or biological samples collected from their participation at any time in the study up until their withdrawal. Data collected up to the date of the withdrawal will be retained in the study to preserve its integrity. Participants who withdraw before completion of the exercise intervention study will be invited and encouraged to participate in all follow-up assessments unless there are safety concerns.

#### Data storage and confidentiality

Identifying information will be kept in locked cabinets in the study coordinator’s office. As such, research assistants will only be provided with a unique number that has been given to each participant upon enrollment in the study. As we will be assessing various parameters that are dependent on age, we will need to retain the participants’ date of birth in order to calculate their age at each assessment period. This is crucial to our data analysis, which will begin once data collection is complete.

Data storage will be in secure servers requiring two-factor authentication. Data are backed up at a remote site for safety in the event of a natural disaster. Participant files will also be stored in a locked cabinet in the study coordinator’s office, to which only the study coordinator and primary investigator possess a key.

#### Dissemination

This RCT is registered with the publicly accessible www.clinicaltrials.gov registry for dissemination and data sharing purposes. RCT results will be published in a high-impact, peer-reviewed journal no more than 12 months after the end of the participant 18-month assessment visits and made freely available for public access within 6 months of publication. We expect to prepare up to ten publications addressing the different facets of this work. We will also present our results at national or international scientific conferences. We will disseminate our study findings on our website, in letters to the editor, participation in online journal clubs, through Alberta Health Services (AHS) newsletters, and internal publications at the University of Calgary and AHS. Similar routes will disseminate the results to other knowledge user team members and partners. Data and biological samples collected during this RCT will be made available to other qualified researchers with the informed consent of participants; data and samples will be de-identified to protect participant confidentiality and privacy.

### Statistical analyses

The primary outcome analysis will use mixed-effects regression analysis to assess the effects of exercise on a composite cognitive score where the aerobic exercise intervention group will be compared to the stretching-toning group while adjusting for the participants’ baseline characteristics (age, sex, MoCA score, years of education, and baseline physical fitness level). The adjusted estimates of the mean change in cognitive composite score for each group will be reported along with the corresponding 95% confidence intervals. Statistical significance will be set at *P* < 0.05, and all tests will be two-sided. The assessment of effect modification (heterogeneity of treatment effect) will be performed with the inclusion of multiplicative interaction terms for age, sex, APOE status, CBF, V̇O_2_max, and sleep quality. A final analysis plan, which includes analyses of the secondary outcomes and strategies for handling missing/incomplete data, will be formalized by the investigators prior to breaking the blind. The data analysis will only begin once data collection is complete.

## Discussion

Results from this RCT will provide additional evidence on the cerebrovascular/physiological, genetic, neuroimaging, sleep, cognitive, and other psychological mechanisms by which 6 months of aerobic exercise may improve cognitive function—in comparison with a stretching-toning intervention—in older adults at elevated risk of ADRD. In addition, data from this study will help determine if any gains seen in cognitive functioning with aerobic exercise are maintained and potentially enhanced by a behavioural support intervention. Given that aerobic exercise is safe, economical, and can be implemented in community settings, our results will have substantial practical importance as they will provide novel evidence that can be used to inform exercise recommendations for the target population (i.e., older individuals at risk of ADRD).

Notable strengths of the current study include a relatively large sample size (*N* = 264), ample post-intervention follow-up period (at 18 months), blinded (in the assessment of outcome measures) RCT design, collection of a multitude of physiological and psychological variables via “gold standard” techniques that may prove to mediate or moderate the relationship among exercise, cognition, and brain health. The ability to characterize how these intervening variables covary with improvements in fitness and cognition due to exercise will provide a wealth of information that may be clinically actionable for those at elevated risk of ADRD due to CVD risk factors. The *Brain in Motion II* Study will individualize aerobic exercise prescriptions based on the fitness levels of each individual (HRR) and track adherence to these training prescriptions via a continuous collection of HR data during exercise sessions. The ability to monitor and measure adherence beyond simple measures of session attendance yields important insights regarding optimal exercise intensity required for cerebrovascular and cognitive benefit from exercise.

While an inactive control group could be considered appropriate from an interval validity standpoint, the decision to compare the aerobic exercise intervention to a stretching-toning one was based on consideration of the limitations associated with the use of an inactive control arm. As most people are aware of the benefits of the exercise, the use of an inactive control group could lead to issues with external validity and may also be considered ethically doubtful for several reasons further discussed in Hecksteden et al. [94]. Additionally, the stretching-toning intervention has been shown to be ineffective or minimally effective compared to aerobic exercise, for several of our outcomes, including some measures of executive function and memory [32], V̇O_2_max [95], BMI [96], and measures of cortical thickness in the frontal cortex [96].

Despite these strengths, this RCT has two main limitations. The nature of the interventions, aerobic exercise and stretching-toning, precludes a double-blind trial design. It is possible that the cognitive outcomes may be, at least partly, influenced by confounding factors, such as socialization offered by the exercise class, participants’ differential expectations for improvement from one type of exercise, and systematic differences in their motivation to improve in fitness. Although these potential confounding variables are difficult to control in a RCT, a previous study showed that participants’ expectations are very unlikely to drive cognitive improvements due to aerobic exercise training [97]. In this study, Stothart et al. (2014) conducted a large survey to examine if people would expect greater improvements in cognitive functioning after aerobic exercise training compared to a control condition (non-aerobic exercise training). Participants who completed the survey expected similar cognitive performance outcomes after aerobic and non-aerobic exercise interventions [97]. It also remains possible that 6 months of aerobic exercise might not be sufficient in length to positively impact cognitive functioning and/or that 1 year of behavioural support is insufficient in length to observe sustained improvements. The results will need to be compared to intermediate findings of those RCTs with longer exercise intervention and/or follow-up periods [35]. Evidence generated by this RCT would be used to inform conversations with primary and community care stakeholders regarding adaptation or incorporation of behavioural support modelled on what we did into the current and future care programmes.

These limitations notwithstanding the Brain in Motion II Study seeks to gain critical insights into the mechanisms by which exercise training improves cognition in older adults at elevated risk of ADRD and stands uniquely situated to do so by examining a large selection of salient physiological and psychological variables. The importance of answers to these questions cannot be overstated given the devastating magnitude of impact that ADRD currently have in the global ageing population.

### Trial status

This trial is ongoing. Enrollment began in January 2017 and is expected to be completed in January 2025. This study reflects the first version of the protocol registered on ClinicalTrials.gov on January 30, 2017 (NCT03035851, https://clinicaltrials.gov/ct2/show/NCT03035851). In response to the COVID-19 pandemic, the research team has adopted extensive additional safety procedures in accordance with the national and provincial public health guidelines, which include an approved Workspace Safety Plan and an ethics modification. Participants enrolled at the time of the public health emergency were given remote support to complete the full duration of the exercise programme. Post-exercise assessments (i.e., 6 months) were completed in the study laboratory on participants who were comfortable with the safety procedures in place after being informed of the steps taken and were willing to come in. At the time of this protocol study submission, enrolment of new participants is still on hold.

## Data Availability

The final trial dataset will be available from the corresponding author on reasonable request.

## Abbreviations

ACSM: American College of Sports Medicine
ACE: Angiotensin-converting enzyme
ADRD: Alzheimer’s disease and related dementias
AHA: American Heart Association
AHS: Alberta Health Services
ANCOVA: Analysis of covariance
APOE: Apolipoprotein E
ASL: Arterial spin labelling
BDNF: Brain-derived neurotrophic factor
BIM II: Brain in Motion II
BIN1: Bridging integrator 1
BISQ: Brain Injury Screening Questionnaire
BMI: Body mass index
BOLD: Blood oxygen level-dependent
CBF: Cerebral blood flow
CLU: Clusterin
CO_2_: Carbon dioxide
COMT: Catechol-*O*-methyl-transferase
CR1: Complement component (3b/4b) receptor 1
CVD: Cardiovascular disease
EQ-5D-5L: EuroQoL Five-Dimension Five-Level Quality of Life Scale
FLAIR: Fluid-attenuated inversion recovery
IDE: Insulin-degrading enzyme
IGF-1: Insulin-like growth factor
HMGCR: 3-Hydroxy-3-methylglutaryl-CoA reductase
HR: Heart rate
HRR: Heart rate reserve
MAC-Q: Memory Assessment Clinic Questionnaire
MoCA: Montreal Cognitive Assessment
MRI: Magnetic resonance imaging
MTHFR: Methylenetetrahydrofolate reductase
TICS-M: Modified Telephone Interview for Cognitive Status
TOMM40: Translocase of outer mitochondrial membrane
PAR-Q+: Physical Activity Readiness Questionnaire
PICALM: Phosphatidylinositol-binding clatherin assembly protein
PROBE: Prospective, Randomized, Open with Blinded End-Points
PSQI: Pittsburgh Sleep Quality Index
REB: Research Ethics Board
RCT: Randomized controlled trial
SAEs: Serious adverse events
V̇O_2_max: Maximal oxygen uptake
